# Supplementary material for: Chromothripsis during telomere crisis is independent of NHEJ, and consistent with a replicative origin
Source: Genome Res. 2019 May;29(5):737–49. doi: 10.1101/gr.240705.118 (PMC6499312; doi:10.1101/gr.240705.118)
Supplement: Supplemental Material [file supp_gr.240705.118_Supplemental_file_1.zip › contigs/annotated_contigs/DB77/contig.2.DB77_length_528_mean_cov_9.62121212121.docx]

**DB77_length_528_mean_cov_9.62121212121**

```
TGAATCACGAGGTCAGGAGTTCGAGACCAGCCTGGCCAACATGGTGAAACCCCGTCTCTACTAAAAATACAAAAAATCAGCTGGGCGTA
 >chr2:14168735-14169021 - E=3e-147
GTGGCAGGTGCCTGTAATCCCAGCTACTCGGGAGGCTGAGGCAGGAGAATCACCTCAACCCGGGAGGCAGAGGTTGCAGTGAGCTGGGA

TTGCACCACTGCATTCCAGCCCTGGCGACAGAGTAAGACTGTGTCTCAGAAAAAAAAAAAAAGAAAACTCAACTTGAGTACGTGTTTAA

GAATTCTCAGGGTG|GA|GAATTCTGAACACACACACACATATGTATATATAATAGCTAGTTATAACAGCTAATGAGAAGACAACCATC
               >chr2:14167554-14167801 - E=5e-137
TACAATAACCATATATACACAGAGATGAGAACAACATGCAGTTCTGATGGAAGAAATACTCCAAAATCTTATTGAGAAACAATGGATTG

TTAAATATAATAGTGTCTTTAATGACGCCATTTTTGAACTTATATCAGTAAACAGATCACCCCAAATTAACAAGTGTAATTTTGT
```
